# Behavior of counterpoise correction in many‐body molecular clusters of organic compounds: Hartree–Fock interaction energy perspective

**DOI:** 10.1002/jcc.26814

**Published:** 2022-02-08

**Authors:** Anh L. P. Nguyen, Ekaterina I. Izgorodina

**Affiliations:** ^1^ School of Chemistry Monash University Victoria Australia

**Keywords:** basis set superposition error, counterpoise correction, Hartree–Fock, many‐body clusters, organic compounds

## Abstract

The counterpoise (CP) correction by Boys and Bernardi has been well accepted as a reliable strategy to account for basis set superposition error (BSSE) in intermolecular complexes. The behavior of the CP correction was thoroughly studied in individual molecules of molecular complexes. This work studies the performance of the CP correction in many‐body clusters including three‐body clusters of organic compounds in the 3B‐69 dataset. Additionally, we used crystal structures of polymorphs of benzene, aspirin, and oxalyl dihydrazide (ODH) to construct a many‐body cluster dataset, abbreviated as the MBC‐36 dataset, consisting of two, four and eight molecules, and 16 molecules in the case of benzene. A series of Dunning's basis sets—cc‐pXZ and aug‐cc‐pXZ (*X* = *D* and *T*)—were used to predict CP‐corrected Hartree–Fock (HF) interaction energies of the 3B‐69 and MBC‐36 datasets. The CP‐corrected interaction energies were found to be basis‐set independent, whereas the non‐CP corrected interaction energies were found not to a follow a smooth exponential fitting as previously found for electronic energies of individual molecules. This observation was attributed to the presence of non‐additive induction forces in some clusters. Two 2 × 2 × 2 supercells of benzene polymorphs were constructed to explore the local nature of BSSE effects. A cut‐off radius of 10 Å was demonstrated to be sufficient to fully recover these effects. Although the behavior of CP correction was found to be non‐conventional in many‐body clusters of organic compounds, the use of a small basis set such as cc‐pVDZ showed excellent performance in the prediction of HF interaction energies.

## Graphical abstract

The basis set superposition error was found to be fully recovered in many‐body clusters of organic compounds when using the conventional counterpoise correction of Boys and Bernardi regardless of basis set.

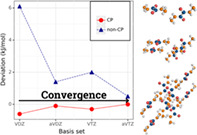

## 1 INTRODUCTION

The accurate prediction of lattice energy in molecular crystals has remained a big challenge in quantum chemistry despite the development of ab‐initio‐ and DFT‐based methods, especially when considering ubiquitous London dispersion forces.^1^, ^2^ Recently, we have proposed a new cost‐effective and accurate methodology for the prediction of lattice energy of molecular crystals.^3^ The methodology is based on the spin‐ratio scaled method modified from the second‐order Møller–Plesset perturbation theory (SRS‐MP2) that was designed to predict correlation interaction energies below 2 kJ mol^−1^ regardless of the interaction type–hydrogen bonding or *π* − *π* stacking.^4^, ^5^ The SRS‐MP2 method was also formulated to eliminate the basis set superposition error (BSSE) that plagues any molecular system consisting of more than one molecule. It must be emphasized that the elimination of BSSE only applies to correlation interaction energy. Since the Hartree–Fock (HF) method forms an essential building block of any wavefunction‐based method such as SRS‐MP2, it is important to understand the influence of the BSSE on HF interaction energies themselves in molecular systems, especially larger clusters consisting of more than two molecules.

Interaction energies, Δ*E* ^INT^ in larger molecular clusters are calculated using the well‐accepted *supermolecule approach*,^6^ in which the entire cluster is treated as a supermolecule and defined as follows:

(1) $$\Delta E^{\mathrm{INT}}=E_{\chi_{M^{1},M^{2},\ldots ,M^{N}}}\left(M^{1},M^{2},\ldots ,M^{N}\right)-\sum_{i=1}^{N}E_{\chi_{M^{i}}}\left(M^{i}\right)$$

where *M* ^*i*^ represents each individual molecule in the cluster, $\chi_{M^{1},M^{2},\ldots ,M^{N}}$ stands for the basis set of the entire supermolecule and $\chi_{M^{i}}$ represents a basis set of the individual molecule *M* ^*i*^.

Basis set superposition error is an artifact of an incomplete basis set and unsurprisingly leads to overbinding.^7^, ^8^ It is well established that in the complete basis set limit, the effect of BSSE vanishes. Several strategies have been proposed in the literature to correct for BSSE, such as the use of a large basis set and extrapolation of total energy to the complete basis set (CBS). Undoubtedly, the most widely used approach is to use the counterpoise (CP) correction scheme suggested by Boys and Bernardi.^9^, ^10^ Within the scheme, the total energy of each individual molecule is calculated within the entire basis set of the cluster, thus resulting in the following definition of the CP‐corrected interaction energy:

(2) $$\Delta E^{\mathrm{CP}‐\mathrm{INT}}=E_{\chi_{M^{1},M^{2},\ldots ,M^{N}}}\left(M^{1},M^{2},\ldots ,M^{N}\right)-\sum_{i=1}^{N}E_{\chi_{M^{1},M^{2},\ldots ,M^{N}}}\left(M^{i}\right)$$

The CP correction has been shown to drastically improve the convergence to the CBS limit using Dunning's basis sets,^8^ thus proving a computationally feasible way of correcting for BSSE.^10^ Contrary to a slow convergence of correlation energy,^11^ the HF convergence occurs relatively fast in interaction energies of molecular dimers, with a quadruple‐*ζ* basis set yielding results close to the complete basis set (CBS) limit.^10^, ^12^, ^13^, ^14^, ^15^, ^16^ In the majority of the published works, the Dunning's correlation‐consistent basis sets (cc‐pVXZ and aug‐cc‐pVXZ with *X* = *D*, *T*, *Q*) have been used. The Boys and Bernardi's correction approach has been also demonstrated to over‐correct for BSSE in some chemical systems due to the imbalance of basis set size describing monomers and corresponding dimers.^8^, ^17^ As shown for dimers of small molecules and atoms,^16^, ^18^, ^19^ the uncorrected HF interaction energy was found to generally overestimate the CBS interaction energy, whereas CP‐corrected interaction energy was found to underestimate the CBS energy. Some exceptions have been found to this rule. For example, in the Be_2_ dimer, both uncorrected and corrected HF energies overestimated the CBS energy.^17^ Various approaches have been proposed to balance the over‐correction,^20^, ^21^ including average uncorrected and CP‐corrected interaction energies.^22^ Increase in basis set size was suggested to be a better way to reduce the effect of BSSE.^23^

Extrapolation of the HF energy to the complete basis set limit has been extensively studied for individual molecules^24^, ^25^, ^26^, ^27^ and has been applied to studying non‐covalent interactions in diverse chemical systems.^28^ Exponential decay of HF energy with increasing basis set was established to best describe the convergence of the HF energy with respect to increasing Dunning's basis sets–both cc‐pVXZ and aug‐cc‐pVXZ (where *X* = *D*, *T*, *Q*). It was also noted that the total HF energies were better fitted with the exponential decay than their binding energies of diatomic molecules.^25^ The error in the latter was not a constant fraction of the total error and differences in converge were observed for the diatomic molecules studied.

To‐date, comprehensive studies on the convergence of the CP correction by Boys and Bernardi have been predominantly performed on molecular dimers.^12^, ^13^, ^14^, ^15^, ^16^ Its behavior in larger clusters consisting of more than two molecules is much less understood. Recently, geometric counterpoise correction has been proposed instead to account for BSSE in periodic calculations.^29^, ^30^, ^31^, ^32^ There is a growing and successful trend in accounting for BSSE using the many‐body expansion and calculating CP‐corrected energies of individual two‐, three‐, and so forth, body interaction energies, instead of performing calculations on the whole cluster.^33^, ^34^ For clusters consisting up to 10 water molecules, the many‐body expansion had errors below 2 kJ mol^−1^ for the prediction of interaction energies using the “gold‐standard” method, CCSD(T)–a coupled cluster theory method incorporating single, double, and perturbative triple excitations.^35^, ^36^ Two‐ and three‐body contributions were sufficient to achieve chemical accuracy (4 kJ mol^−1^).

Řezáč et al. published a benchmark database consisting of intermolecular clusters of three molecules, that is, trimers.^37^ The database, termed 3B‐69, was constructed from well‐studied crystal structures of 23 organic compounds such as water, formaldehyde, acetonitrile, and so forth. Sixty‐nine trimers with three different configurations for each compound were extracted from experimental crystal structures that were additionally relaxed using periodic boundary conditions. Three‐body contributions arising from induction and dispersion were found to contribute between 4% and 20%. Considering only the three‐body energy, two‐thirds of the studied trimers had contributions between 5% and 25% from London dispersion forces toward it, whereas one third had more than 25% contribution. Errors in HF interaction energies calculated with the aug‐cc‐pVQZ basis set were established to only moderately correlate with the three‐body dispersion component, thus exhibiting non‐negligible polarization errors.

Kamiya et al.^38^ proposed an effective alternative approach to incorporating basis set superposition error in molecular clusters based on the truncated Valiron–Mayer function CP (VMFC) scheme. The approach relies on the truncation of the many‐body expansion after two‐ and three‐body terms and includes high‐order Coulomb terms that are not accounted for in the original VMFC scheme and adopted from the Fragment Molecular Orbital approximation. Errors below 0.1 kcal mol^−1^ were found for clusters of water and hydrogen fluoride.

In recent years a number of groups^33^, ^34^, ^35^, ^39^ have focused on studying the effect of BSSE on the prediction of binding energies of many‐body clusters, whose electronic energies are approximated within the many‐body expansion framework through a series of one‐body, two‐body, three‐body, and so forth, interactions. Compared to previous studies, the HF electronic energies of the many‐body clusters in our work are not decomposed using a fragment‐based approach such as the MBE in our work. Instead, these are calculated the conventional way to investigate the behavior of the CP‐corrected interaction energies in many‐body clusters of organic compounds as originally proposed by Boys and Bernardi.

This study was inspired by the work of Řezáč et al.^37^ The 3B‐69 database was taken to analyze the CP corrected interaction energies calculated with HF and a series of Dunning's correlation consistent basis sets–cc‐pVXZ and aug‐cc‐pVXZ (*X* = *D*, *T*, *Q*). In addition, crystal structures of the well‐studied polymorphs of benzene (BZ), aspirin (ASP) and oxalyl dihydrazide (ODH) were taken to extract many‐body clusters consisting of two (dimers), four (tetramers), eight (octamers), and 16 (hexadecamers) molecules. Two polymorphs of benzene (forms I and II) and aspirin (forms I and II) were selected in this study as well as five polymorphs of ODH (forms *α*, *β*, *δ*, *γ*, and *ϵ*). CP‐corrected HF interaction energies of these clusters were analyzed against increasing cluster size (i.e., increasing number of molecules in the cluster) and increasing basis set in the series of Dunning's cc‐pVXZ and aug‐cc‐pVXZ basis sets (*X* = *D*, *T*, *Q*).

## 2 METHODOLOGY

### 2.1 Datasets

**FIGURE 1:**
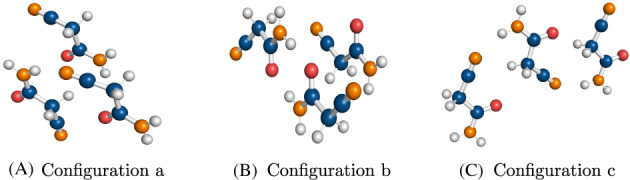
Structures of three distinct configurations of 2‐cyanoacetamide trimers

In this work, we deployed the three‐body dataset, 3B‐69, created by Řezáč et al.^37^ From each of the 23 organic crystals that were additionally relaxed using periodic boundary conditions, three different configurations of a three‐body cluster were extracted. This resulted in 69 trimers overall. The crystal structures used to build the dataset consisted of small organic molecules containing C, H, O, and N atoms, with varying contributions from London dispersion forces and hydrogen bonding interactions as well as three‐body induction and dispersion contributions. These three‐body clusters were divided into three categories depending on the dispersion contribution to the three‐body energy: low (below 5% of dispersion), medium (between 5% and 25% of dispersion) and high (above 25% of dispersion). On several occasions, the crystal structure of the same compound produces clusters of low, medium, and high dispersion at the same time. Figure 1 shows an example of three distinct configurations of 2‐cyanoacetamide trimers. Compound names of the whole 3B‐69 dataset are provided in the Supporting Information (see Table S1).

**FIGURE 2:**
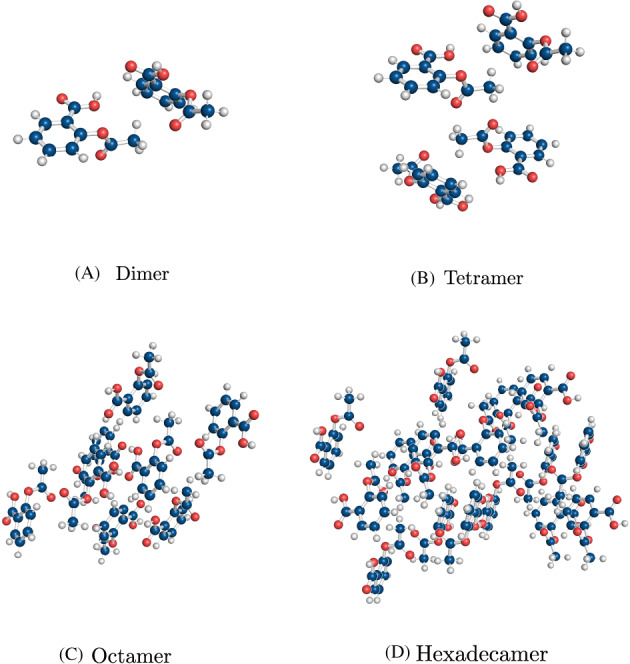
Many‐body clusters extracted from the crystal structure of aspirin form I. Carbon atoms are depicted in blue, hydrogen atoms in white and oxygen atoms in red

In this study, we generated another many‐body cluster dataset, termed MBC‐36 due to 36 many‐body clusters, based on the crystal structures of benzene (BZ), aspirin (ASA), and oxalyl dihydrazide (ODH). Each of these compounds exists in several polymorphs. Oxalyl dihydrazide has five polymorphs: *α*, *β*, *δ*, *γ*, and *ϵ*, while both aspirin and benzene have two well‐known polymorphs – forms I and II. For each polymorph, many‐body clusters consisting of two, four, and eight molecules were extracted from the corresponding crystal structure. The cluster composition was randomly selected not to favor any particular intermolecular interaction. For form I of benzene, an additional 16‐molecule cluster was also included. Figure 2 shows an example of many‐body clusters extracted from the crystal structure of aspirin in form I. Many‐body clusters of all the polymorphs can be found in the Supporting Information (see Figures S1–S9).

### 2.2 Theoretical methodology

The interaction energies across both datasets were calculated with the HF method and five Dunning's correlation consistent basis sets: cc‐pVDZ, aug‐cc‐pVDZ, cc‐pVTZ, aug‐cc‐pVTZ, and cc‐pVQZ. The application of aug‐cc‐pVQZ was not computationally feasible for larger many‐body clusters, especially octamers and hexadecamers. The BSSE was accounted for with the CP correction scheme by Boys and Bernardi.^9^ The CP‐corrected HF interaction energies performed with the cc‐pVQZ basis set were taken as the benchmark in this work. All calculations were carried out in Psi4 v1.1^40^ using the resolution of identity (RI) approximation.

CP‐ and non‐CP corrected interaction energies were analyzed in the conventional way by recalculating these per molecule. Further in the text, when discussing interaction energies of many‐body clusters, we refer to *interaction energies per molecule*.

Root mean square deviations (RMSDs), raw deviations, and mean unsigned errors (MUEs) were used to analyze the convergence of the uncorrected and CP‐corrected HF interaction energies of the 3B‐69 and MBC‐36 datasets.

(3) $$\text{RMSD}=\sqrt{\frac{\sum_{i=1}^{N}{\left(\Delta E_{\mathrm{HF}/\mathrm{cc}‐\text{pVQZ}}^{\mathrm{CP}‐\mathrm{INT}}-\Delta E_{\mathrm{HF}/\left(\mathrm{aug}\right)‐\mathrm{cc}‐\text{pVXZ}}^{\left(\mathrm{CP}\right)‐\mathrm{INT}}\right)}^{2}}{N}}$$

(4) $$\mathrm{MUE}=\frac{\sum_{i=1}^{N}\left|\Delta E_{\mathrm{HF}/\mathrm{cc}‐\text{pVQZ}}^{\mathrm{CP}‐\mathrm{INT}}-\Delta E_{\mathrm{HF}/\left(\mathrm{aug}\right)‐\mathrm{cc}‐\text{pVXZ}}^{\left(\mathrm{CP}\right)‐\mathrm{INT}}\right|}{N}$$

where Δ*E* ^CP‐INT^ is the CP‐corrected interaction energy and *N* is the number of data points used, *X* denotes *D* and *T* for (aug)‐cc‐pVDZ and (aug)‐cc‐pVTZ. Further in the text, the CP abbreviation stands for CP corrected interaction energy, whereas the non‐CP abbreviation implies non‐corrected interaction energy.

Raw deviations were calculated by taking the difference between the benchmark interaction energy, $\Delta E_{\mathrm{HF}/\mathrm{cc}‐\text{pVQZ}}^{\mathrm{CP}‐\mathrm{INT}}$, and interaction energy of a given basis set, $\Delta E_{\mathrm{HF}/\left(\mathrm{aug}\right)‐\mathrm{cc}‐\text{pVXZ}}^{\left(\mathrm{CP}\right)‐\mathrm{INT}}$ (where *X* denotes *D* and *T* for (aug)‐cc‐pVDZ and (aug)‐cc‐pVTZ). Interaction energies were taken as per molecule quantities.

**FIGURE 3:**
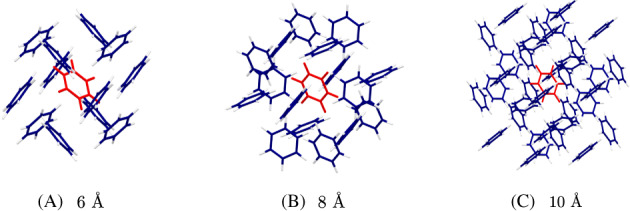
Benzene clusters that were used to calculate CP correction with three cut‐off radii with the 2 × 2 × 2 cell. The reference molecule is colored in red

By default, the CP correction is calculated using the monomer energies in the cluster basis set (see Equation (2)). It has been hypothesized that the basis set superposition error is a short‐ranged effect, with the inclusion of the basis set functions of the entire system may not be necessary. For example, exclusion of diffuse functions on hydrogen atoms in dimers of ammonia, water, and hydrogen fluoride did not affect geometry optimization with the CP‐corrected MP2 method.^41^ In this study, we investigated the convergence of the CP correction with respect to the distance between a reference monomer and other monomers in the system. For this purpose, we constructed a 2 × 2 × 2 unit‐cell system from the crystal structure of benzene in form I. The CP correction was calculated with respect to a randomly selected reference molecule in the middle of the cluster. Basis functions of the monomers within a given radius were included in the calculation. The center of mass of the reference benzene molecule was chosen as the reference point for the cut‐off radius. Three cut‐off radii were used in the study: 6, 8, and 10 Å (see Figure 3).

## 3 RESULTS AND DISCUSSIONS

### 3.1 Three‐body 3B‐69 dataset

**FIGURE 4:**
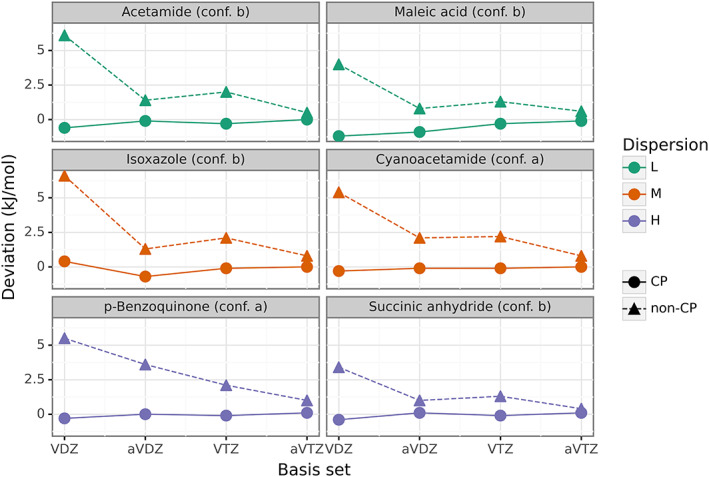
Behavior of raw deviations (in kJ mol^−1^) of non‐CP and CP‐corrected HF interaction energies with increasing basis set for six complexes of the 3B‐69 dataset. CP‐corrected HF/cc‐pVQZ interaction energies were used as the benchmark

The 3B‐69 dataset provides diverse structures of molecular complexes of small organic molecules. Figure 4 shows the behavior of raw deviations, with increasing basis set with respect to the CP corrected HF interaction energies with cc‐pVQZ. As an example, six complexes from three categories mentioned above of low, medium, and high dispersion are presented. In this study, we decided to calculate deviations, interaction energies as well as statistical measures per molecule, since thermodynamic quantities such as molar entropy, lattice energy, and so forth, are usually given on the per mole basis. Therefore, the comparison of the magnitude of deviations calculated per molecule are directly comparable to these thermodynamic quantities. The unsigned deviations calculated with different basis sets for each complex in the 3B69 are given in the Tables S2–S5 in the Supporting Information.

In line with previously observed trends, there is a similar behavior found in non‐CP corrected energies in the 3B‐69 dataset. As originally reported for electronic energies of single molecules, non‐CP corrected energies usually approach the complete basis set energy from above through overbinding. In the case of the three‐body clusters, non‐CP corrected interaction energies were also found to slightly overbind, thus resulting in positive deviations as depicted in Figure 4. Deviations for CP‐corrected energies are rather linear and negligible. These also appear to be predominantly basis set independent. The behavior of the non‐CP energies is not consistently smooth as opposed to an exponential decay observed for electronic energies of single molecules with increasing basis set. It is obvious that this trend changes as we transition to many‐body complexes. This can be attributed to non‐additive induction forces present in the 3B‐69 dataset. It was shown by Řezáč et al.^37^ that three‐body induction effects were important even in such simple systems, varying to a large degree even within the same organic compound. The observation also indicates that an exponential fitting can no longer be applied to three‐body complexes.

In terms of the absolute deviations, RMSD values of the CP corrected energies are already under 1 kJ mol^−1^ for all the three‐body complexes regardless of their interaction type and basis set. In the original work by Řezáč et al.,^37^ the complexes were divided into three categories based on the three‐body energy: low (below 5% of dispersion), medium (between 5% and 25% of dispersion) and high (above 25% of dispersion). As long as the CP correction is included, the HF interaction energy is ensured to be nearly BSSE‐free and is found to deviate with sub‐kJ mol^−1^ accuracy with respect to the benchmark, as displayed in Table 1. Cc‐pVDZ already offers excellent accuracy. As expected, non‐CP corrected interaction energies produce larger deviations from the benchmark. Not surprisingly, cc‐pVDZ showed the largest RMSD of 5.4 kJ mol^−1^ as well as a maximum error of 9.7 kJ mol^−1^. Aug‐cc‐pVTZ appears to recover majority of the BSSE effects without the need to use the CP correction producing an RMSD of 0.7 kJ mol^−1^.

**TABLE 1:** Root mean square deviations (RMSD), mean unsigned errors (MUEs) and absolute maximum deviations (Max) (in kJ mol^−1^) for non‐CP and CP‐corrected HF interaction energies in the 3B‐69 dataset. All energies were calculated per molecule. CP‐corrected HF/cc‐pVQZ were taken as the benchmark

Δ*E* ^INT^	Basis set	RMSD	MUE	Max
CP corrected	cc‐pVDZ	0.6	0.5	1.7
	aug‐cc‐pVDZ	0.3	0.2	0.9
	cc‐pVTZ	0.2	0.1	0.4
	aug‐cc‐pVTZ	0.1	0.1	0.3
Non‐CP corrected	cc‐pVDZ	5.4	5.0	9.7
	aug‐cc‐pVDZ	2.1	1.9	4.8
	cc‐pVTZ	1.9	1.8	3.5
	aug‐cc‐pVTZ	0.7	0.6	1.2

### 3.2 Many‐body clusters

This section analyses the influence of CP correction on the HF interaction energies in molecular clusters of organic compounds with increasing cluster size from two molecules to eight molecules and in the case of benzene (form I) to 16 molecules.

**FIGURE 5:**
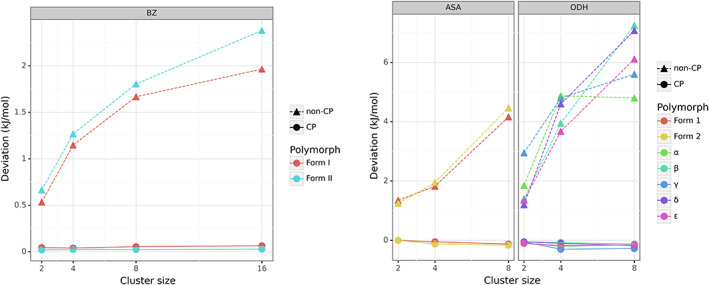
Behavior of average raw deviations (kJ mol^−1^) of non‐CP and CP‐corrected HF/cc‐pVTZ interaction energies with increasing cluster size and basis set in the MBC‐36 complexes. CP corrected HF/cc‐pVQZ interaction energies were taken as the benchmark

As in previous sections, similar diagrams for raw deviation distributions are displayed in Figure 5, with cc‐pVTZ being given as an example here. The trend for the rest of the basis sets can be found in the Supporting Information (see Figures S10–S15). On each diagram, the *x*‐axis manifests the cluster size. It can be clearly seen that the CP corrected interaction energies result in interaction energies well within 1 kJ mol^−1^ regardless of the basis set size used. Non‐CP corrected interaction energies systematically overbind with increasing cluster size. From the analysis of Figure 5, one can clearly conclude that the behavior of non‐CP corrected interaction energies is very dependent on the cluster nature. Benzene clusters for both form I and form II demonstrate a smooth behavior in non‐CP corrected HF energies with increasing cluster size. It has been recently shown that three‐body effects are rather negligible in crystal structures of both forms.^3^ Therefore, such smooth behavior is expected. With introduction of non‐additive interactions such as induction forces present in hydrogen‐bonded clusters of ODH and to some extent in aspirin, the non‐CP corrected HF energies behave differently, no longer exhibiting a smooth behavior with increasing cluster size. In particular, the aspirin clusters showed a slow increase in Δ*E* ^INT^ deviations in smaller clusters of two and four molecules, whereas in the ODH clusters a steep increase in Δ*E* ^INT^ deviations was observed going from two to four molecule clusters. The CP‐corrected energies were found to slightly underbind well within 0.3 kJ mol^−1^, further indicating that the CP correction almost completely recovers BSSE effect with Dunning's basis sets. These findings clearly identify the non‐additive nature of BSSE effects and highlight the danger of using varying fitting protocols for non‐CP corrected HF interaction energies of large‐scale clusters.

Table 2 presents RMSD, MUE, and maximum error values for clusters of benzene, aspirin, and ODH calculated with the cc‐pVTZ basis set. The rest of the basis sets can be found in the Supporting Information (see Tables S6–S17). For the largest clusters deviations of up to 19.8 kJ mol^−1^ were observed for one of the eight‐molecule clusters of ODH when combined with cc‐pVDZ. It can be clearly seen that for each system the behavior of non‐CP corrected energies is rather unique. This is most likely the manifestation of varying interaction types. In the case of aspirin, the clusters contain hydrogen‐bonded dimers and C—H⋯*π* interactions. On the other hand, the clusters of ODH are entirely driven by hydrogen bonding, whereas in the benzene clusters *π*–*π* stacking interactions are the main interaction type. The observed trends strongly suggest that the application of any sort of fitting to non‐CP corrected energies will be unique to each compound and cannot be transferred between clusters of varying nature. For the 16‐molecule benzene clusters, non‐CP Δ*E* ^INT^ energies deviated up to 6.5 and 13.3 kJ mol^−1^ with cc‐pVDZ and aug‐cc‐pVDZ, respectively, with deviations reducing to below 2.5 kJ mol^−1^ for the larger basis sets. The eight‐molecule clusters of ODH and aspirin can produce deviations of up to 19.8 and 13.0 kJ mol^−1^ for cc‐pVDZ, respectively. Surprisingly, the CP‐corrected interaction energies did not appear to depend on basis set, showing negligible errors below 0.3 kJ mol^−1^. Based the presented result, we can confidently conclude that the accurate calculation of HF interaction energies of clusters of organic molecules can be performed as long as these are BSSE corrected.

**TABLE 2:** Root mean square deviations (RMSD), mean unsigned errors (MUEs) and absolute maximum deviations (Max) (in kJ mol^−1^) for non‐CP and CP‐corrected HF/cc‐pVTZ interaction energies in the MBC dataset. All energies were calculated per molecule. CP‐corrected HF/cc‐pVQZ were taken as the benchmark

Compound	Δ*E* ^INT^	Cluster size	RMSD	MUE	Max
Benzene	CP corrected	2	0.04	0.03	0.05
		4	0.03	0.03	0.04
		8	0.04	0.04	0.06
		16	0.05	0.05	0.07
	Non‐CP corrected	2	0.6	0.6	0.6
		4	1.2	1.2	1.3
		8	1.7	1.7	1.8
		16	2.2	2.2	2.4
Aspirin	CP corrected	2	0.00	0.00	0.00
		4	0.10	0.09	0.13
		8	0.15	0.14	0.16
	Non‐CP corrected	2	1.3	1.3	1.4
		4	1.9	1.9	2.0
		8	4.3	4.3	4.3
ODH	CP corrected	2	0.06	0.06	0.09
		4	0.18	0.17	0.30
		8	0.18	0.17	0.27
	Non‐CP corrected	2	1.9	1.8	3.0
		4	4.4	4.4	4.9
		8	6.2	6.2	7.2

### 3.3 Behavior of CP correction with cut‐off radius

It is widely understood that BSSE is largely a local effect waning with distance. In this study, we decided to demonstrate a potential application of this strategy in larger clusters by calculating CP corrections with several radius cut‐offs with respect to the reference monomer. The radius cut‐off is used to identify which adjacent molecules to a given molecule are taken into account when computing its CP correction. If the center of mass of a molecule is separated from that of the reference molecule within the cut‐off value, this molecule is included in the basis set of the reference molecule in the energy calculation.

**FIGURE 6:**
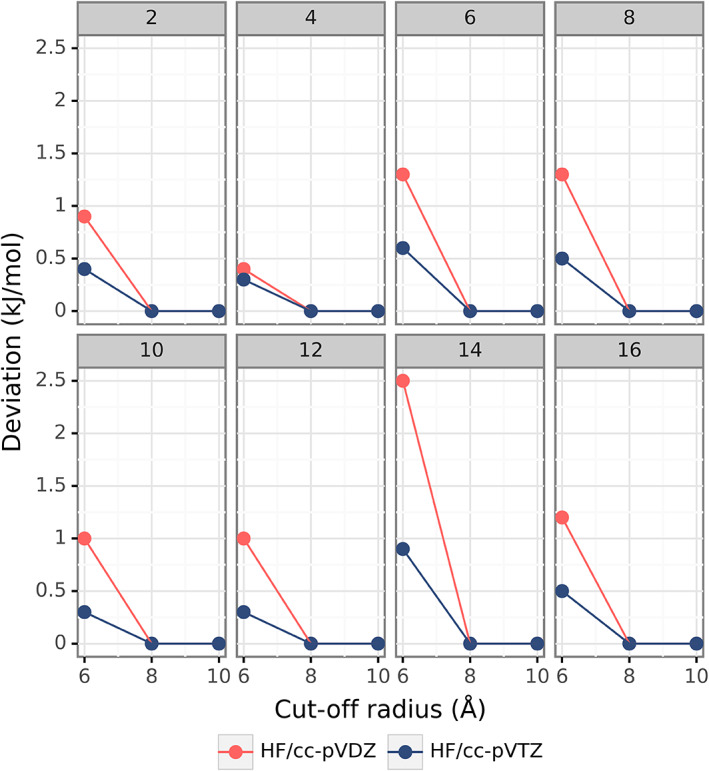
Raw deviations (in kJ mol^−1^) of CP‐corrected HF electronic energies of individual monomers in the 16‐molecule benzene cluster with increasing cut‐off radius. CP‐corrected HF/cc‐pVQZ electronic energy for each monomer was taken as the benchmark

For the 16‐molecule cluster of benzene (form I), Figure 6 displays behavior of CP‐corrected electronic energies of individual monomers with increasing cut‐off radius from 6 to 8 and 10 Å(for raw electronic energies see Tables S18–S21 in the Supporting Information). Two basis sets were employed here, cc‐pVDZ and cc‐pVTZ. As one can see, the deviation of CP‐corrected HF electronic energies decreases with increasing cut‐off radius, nearly vanishing at 10 Å regardless of the basis set. As expected, at the shortest cut‐off radius of 6 Å, the absolute deviation depends on the position of the monomer in the cluster. The deviations calculated cc‐pVDZ did not exceed 3 kJ mol^−1^, whereas, when cc‐pVTZ was used the deviations decreased to below 1 kJ mol^−1^.

**FIGURE 7:**
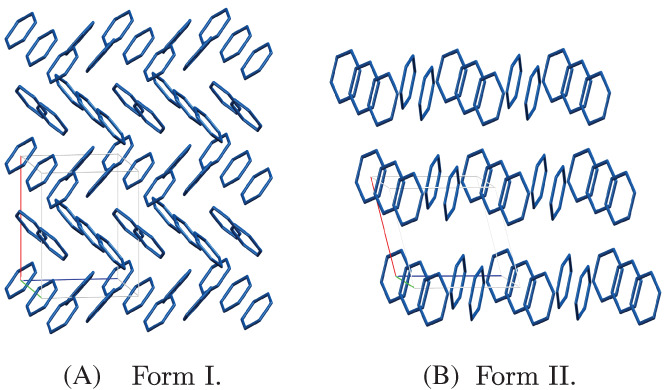
Structures of the 2 × 2 × 2 supercells of benzene form I and II

The 2 × 2 × 2 supercells were constructed from the unit cells reported for the crystal structures of both forms of benzene (see Figure 7). There were 63 monomers present in the supercell of form I and 39 monomers in the supercell of form II.

The HF interaction energies of the two supercells were calculated with both cc‐pVDZ and cc‐pVTZ basis sets. The CP‐correction was used with a 10 Å cut‐off radius for each monomer. Through the use of this cut‐off, much computational time was saved, with each CP correction calculation taking minutes instead of hours. Thus calculated HF interaction energies are shown in Table 3.

**TABLE 3:** CP corrected HF interaction energies (in kJ mol^−1^) of the 2 × 2 × 2 supercells of benzene calculated with cc‐pVDZ and cc‐pVTZ and a cut‐off radius of 10 Å for CP correction. The energies were calculated per molecule

Basis set	Form I	Form II
cc‐pVDZ	5.1	14.1
cc‐pVTZ	5.3	14.0

Analysis of Table 3 reveals that CP‐corrected HF interaction energies of the two supercells of benzene calculated with cc‐pVDZ and cc‐pVTZ agree with each other within 0.2 kJ mol^−1^. Furthermore, the interaction energy of form I with cc‐pVDZ completely aligns with the result calculated using the CP‐correction in the full basis set, 5.1 kJ mol^−1^. This is an excellent finding, further highlighting the local nature of BSSE that can be recovered using a 10 Å cut‐off radius in larger clusters of organic compounds.

## 4 CONCLUSIONS

In this study, we investigated the behavior the CP correction by Boys and Bernardi in many‐body complexes of organic compounds included in two datasets, 3B‐69 and MBC‐36. As expected, the non‐CP HF interaction energies were found to overestimate interaction energy, with errors increasing with increasing number of molecules in a cluster. In both datasets the non‐CP interaction energy did not exhibit a smooth exponential behavior with increasing basis set, prohibiting the application of an exponential fitting that was demonstrated to work for single molecules and dimers. This observation was attributed to the presence of non‐additive forces in many‐body clusters. In the MBC‐36 dataset, clusters were found to exhibit varying types of intermolecular forces from hydrogen bonding to *π*–*π* stacking, thus leading to distinct trends in non‐CP HF interaction energies. The latter demonstrated a smooth behavior with increasing basis set, whereas the presence of hydrogen bonding resulted in a varied behavior. In some cases a decrease in non‐CP energy was observed going from smaller‐ to medium‐sized clusters. The application of the CP correction by Boys and Bernardi significantly improves the quality of HF interaction energy with respect to the benchmark results. RMSD values were found to be well within 1 kJ mol^−1^ across all basis est and cluster sizes. Therefore, even a small basis set such as cc‐pVDZ can already provide excellent accuracy as long as the HF interaction energies are corrected for BSSE using the CP scheme. Additionally, we studied the local nature of BSSE demonstrating that a cut‐off radius of 10 Å was already sufficient to fully account for BSSE. This strategy was successfully applied in the 2 × 2 × 2 supercells of two polymorphs of benzene—forms I and II, producing errors below 0.2 kJ mol^−1^. This strategy will allow us to significantly reduce the cost of HF calculations for large‐scale chemical systems.

## Supporting information


**Appendix S1**: Supporting information.

## Data Availability

The data that supports the findings of this study are available in the Supporting Information of this article.
