# Supplementary material for: Behavior of counterpoise correction in many‐body molecular clusters of organic compounds: Hartree–Fock interaction energy perspective
Source: J Comput Chem. 2022 Feb 8;43(8):568–76. doi: 10.1002/jcc.26814 (PMC9303541; doi:10.1002/jcc.26814)
Supplement: Supplementary file 1 — Appendix S1: Supporting information. [file JCC-43-568-s001.pdf]

Supplementary Information to accompany

Behaviour of counterpoise correction in many-body molecular clusters of organic compounds: Hartree-Fock interaction energy perspective.

Anh Luu Phuong Nguyen, Ekaterina I. Izgorodina.

Contents

1 3B69 dataset **2**
1.1 Structures 2
1.2 Raw deviations 2

2 MBC-36 dataset **8**
2.1 Structures 8
2.1.1 Aspirin 9
2.1.2 Benzene 9
2.1.3 Oxalyl dihydrazide 10
2.2 Statistical errors 12
2.3 Raw deviations 16

3 CP correction with cut-off radius **18**

1 3B69 dataset

1.1 Structures

Table S1 Compound names included in the 3B69 dataset.

Structure number	Name
01	Water
02	Formaldehyde
03	Methanol ethyne
04	Acetonitrile
05	Nitromethane
06	Acetic acid
07	Oxalic acid
08	Vinylene carbonate
09	Acetamide
10	Imidazole
11	Isoxazole
12	Pyrazole
13	Triazine
14	Cyanoacetamide
15	Cyanoguanidine
16	Triazolidinedione
17	Oxazolidinone
18	Succinic anhydride
19	Benzene
20	Maleic acid
21	p-Benzoquinone
22	Uracil
23	Cyclobutylfuran

Each structure has three different configurations, which are symbolised with a, b and c. For example, the corresponding complexes of water are labeled as 01a, 01b and 01c.

1.2 Raw deviations

Table S2 Raw deviations (in kJ·mol^{-1}) of non-CP and CP-corrected HF/cc-pVDZ interaction energies of the 3B69 complexes. CP-corrected HF/cc-pVQZ interaction energies were used as the benchmark. The dispersion type shows the contribution of dispersion in the interaction energy: L = low, M = medium, H= high.

Structure	Dispersion type	CP	non-CP
01a	L	0.1	6.7
01b	L	-1.1	5.8
01c	L	0.7	9.7
02a	L	-0.3	1.6
02b	M	-0.2	2.7
02c	M	-0.5	2.8
03a	L	0.1	6.6
03b	H	-0.1	1.1

Table S2 continued from previous page

03c	M	-0.1	4.2
04a	M	-0.5	1.5
04b	L	-0.2	1.7
04c	L	-0.3	0.8
05a	M	0.8	6
05b	M	1.2	7
05c	L	0.5	4.3
06a	L	-0.6	5.2
06b	L	-1	6.2
06c	H	-0.6	6.3
07a	M	0.5	5.8
07b	L	0.1	6
07c	L	0.1	6
08a	L	1.4	5.2
08b	M	0.7	4.8
08c	L	1.7	6.6
09a	H	-0.6	6.8
09b	L	-0.6	6.1
09c	L	-0.5	5.7
10a	M	0.2	4.7
10b	M	0	2.8
10c	L	0.5	5.2
11a	H	0.4	7.2
11b	M	0.4	6.6
11c	L	1	6
12a	L	0.6	5.6
12b	H	0	2.8
12c	L	0.2	3.8
13b	H	0.3	4.3
13c	H	0	4.1
14a	M	-0.3	5.4
14b	H	-0.5	6.7
14c	L	-0.5	4.3
15a	L	0.3	6.5
15b	H	-0.9	6.5
15c	M	-0.6	3.9
16a	M	1.2	8.2
16b	L	-1.2	6.6
16c	M	0.2	7.4
17a	L	-0.3	5.8
17b	M	-0.4	6.8
17c	L	-0.1	5.2
18a	M	0.4	7
18b	H	-0.4	3.4
18c	M	0.8	5.6
19a	H	0.2	2.1
19b	H	0.2	2.1
19c	H	0.2	2
20a	L	-0.8	7.5

Table S2 continued from previous page

20b	L	-1.2	4
20c	M	0.6	7.2
21a	H	-0.3	5.5
21b	H	0.1	5.1
21c	H	-0.2	3.5
22a	H	-0.2	8
22b	M	1	6.5
22c	M	0.6	4.6
23a	H	0.2	2.8
23b	H	0.4	3.5
23c	H	-0.1	3.3

Table S3 Raw deviations (in kJ·mol^{-1}) of non-CP and CP-corrected HF/cc-pVTZ interaction energies of the 3B69 complexes. CP-corrected HF/cc-pVQZ interaction energies were used as the benchmark. The dispersion type shows the contribution of dispersion in the interaction energy: L = low, M = medium, H= high.

Structure	Dispersion type	CP	non-CP
01a	L	-0.1	2.5
01b	L	-0.4	2.3
01c	L	0.2	3.5
02a	L	-0.1	0.6
02b	M	-0.1	0.9
02c	M	-0.1	1
03a	L	-0.2	2.1
03b	H	0	0.6
03c	M	-0.2	1.6
04a	M	-0.1	0.6
04b	L	-0.1	0.6
04c	L	-0.1	0.4
05a	M	0.2	2.2
05b	M	0.2	2.6
05c	L	0.1	1.9
06a	L	-0.2	1.9
06b	L	-0.2	2
06c	H	-0.2	2.3
07a	M	-0.1	2.2
07b	L	-0.1	1.7
07c	L	-0.1	1.8
08a	L	0.3	1.5
08b	M	0.2	1.8
08c	L	0.2	1.8
09a	H	-0.2	2.4
09b	L	-0.3	2
09c	L	-0.2	1.9
10a	M	0	1.7
10b	M	0	1.5
10c	L	-0.1	1.7
11a	H	-0.1	2.6

Table S3 continued from previous page

11b	M	-0.1	2.1
11c	L	0.1	1.7
12a	L	-0.1	1.9
12b	H	0	1.3
12c	L	0	1.5
13b	H	0.1	1.5
13c	H	0	1.5
14a	M	-0.1	2.2
14b	H	-0.2	2.4
14c	L	-0.1	1.5
15a	L	0.1	2.3
15b	H	-0.3	2.5
15c	M	-0.1	2
16a	M	0.2	2.9
16b	L	-0.4	2
16c	M	-0.1	2.5
17a	L	-0.3	1.7
17b	M	-0.2	2.6
17c	L	0	2.1
18a	M	0	2.4
18b	H	-0.1	1.3
18c	M	0.1	1.9
19a	H	0	0.8
19b	H	0	0.8
19c	H	0	0.8
20a	L	-0.2	3
20b	L	-0.3	1.3
20c	M	0.1	3.2
21a	H	-0.1	2.1
21b	H	-0.2	1.6
21c	H	-0.1	1.6
22a	H	-0.3	2.1
22b	M	0.2	2.7
22c	M	0.1	1.8
23a	H	0	1.2
23b	H	-0.1	1.1
23c	H	0	1.5

Table S4 Raw deviations (in kJ·mol^{-1}) of non-CP and CP-corrected HF/aug-cc-pVDZ interaction energies of the 3B69 complexes. CP-corrected HF/cc-pVQZ interaction energies were used as the benchmark. The dispersion type shows the contribution of dispersion in the interaction energy: L = low, M = medium, H= high.

Structure	Dispersion type	CP	non-CP
01a	L	-0.3	0.5
01b	L	0	1
01c	L	-0.4	0.7
02a	L	0.1	0.5
02b	M	0.1	0.8

Table S4 continued from previous page

02c	M	0.1	0.9
03a	L	-0.5	0.6
03b	H	0	0.6
03c	M	-0.2	0.6
04a	M	0	0.9
04b	L	0	0.9
04c	L	0.1	0.7
05a	M	0.1	1.7
05b	M	0.1	1.8
05c	L	0.1	1.3
06a	L	-0.3	1.2
06b	L	-0.6	1.1
06c	H	-0.2	1.8
07a	M	0.1	2.5
07b	L	-0.4	1.9
07c	L	-0.3	2
08a	L	0	1.5
08b	M	0.1	2.4
08c	L	0	2.1
09a	H	-0.2	1.7
09b	L	-0.1	1.4
09c	L	-0.2	1.3
10a	M	0	1.8
10b	M	0	1.5
10c	L	-0.1	1.1
11a	H	-0.6	2.7
11b	M	-0.7	1.3
11c	L	-0.7	1.1
12a	L	-0.3	1.9
12b	H	0.1	2.1
12c	L	0	1.6
13b	H	0.1	1.1
13c	H	0.1	1.5
14a	M	-0.1	2.1
14b	H	-0.2	1.9
14c	L	-0.1	1.7
15a	L	-0.4	1.7
15b	H	-0.1	2
15c	M	-0.2	1.1
16a	M	0	3.8
16b	L	-0.5	2.2
16c	M	-0.2	2.9
17a	L	-0.1	1.7
17b	M	0	2.6
17c	L	0.1	2.4
18a	M	0	2.5
18b	H	0.1	1
18c	M	-0.1	2
19a	H	0.1	3

Table S4 continued from previous page

19b	H	0.1	3.1
19c	H	0	3.2
20a	L	-0.4	3.2
20b	L	-0.9	0.8
20c	M	0	4.5
21a	H	0	3.6
21b	H	0.2	2.2
21c	H	0	2.8
22a	H	0	2.9
22b	M	0	4.8
22c	M	0	2.9
23a	H	0	3.3
23b	H	0	2.2
23c	H	0	2.8

Table S5 Raw deviations (in kJ·mol^{-1}) of non-CP and CP-corrected HF/aug-cc-pVTZ interaction energies of the 3B69 complexes. CP-corrected HF/cc-pVQZ interaction energies were used as the benchmark. The dispersion type shows the contribution of dispersion in the interaction energy: L = low, M = medium, H= high.

Structure	Dispersion type	CP	non-CP
01a	L	-0.1	0.2
01b	L	0.2	0.5
01c	L	-0.3	0
02a	L	0.1	0.2
02b	M	0.1	0.3
02c	M	0.1	0.3
03a	L	0	0.3
03b	H	0	0.2
03c	M	0	0.2
04a	M	0	0.3
04b	L	0	0.3
04c	L	0	0.3
05a	M	0	0.5
05b	M	0	0.6
05c	L	0	0.4
06a	L	0	0.5
06b	L	-0.1	0.6
06c	H	0	0.7
07a	M	0.2	1.1
07b	L	-0.2	0.6
07c	L	-0.1	0.7
08a	L	-0.1	0.4
08b	M	0	0.8
08c	L	-0.1	0.6
09a	H	-0.1	0.6
09b	L	0	0.5
09c	L	-0.1	0.4
10a	M	0	0.6

Table S5 continued from previous page

10b	M	0	0.4
10c	L	0	0.5
11a	H	0	1.1
11b	M	0	0.8
11c	L	-0.1	0.6
12a	L	0	0.6
12b	H	0	0.5
12c	L	0	0.4
13b	H	0	0.4
13c	H	0	0.5
14a	M	0	0.8
14b	H	-0.1	0.7
14c	L	0	0.7
15a	L	-0.2	0.5
15b	H	0.2	0.9
15c	M	0	0.5
16a	M	0	1
16b	L	-0.1	0.8
16c	M	0	0.9
17a	L	0	0.6
17b	M	0	0.7
17c	L	0	0.6
18a	M	0.1	0.9
18b	H	0.1	0.4
18c	M	0	0.8
19a	H	0	0.5
19b	H	0	0.5
19c	H	-0.1	0.5
20a	L	0	1.1
20b	L	-0.1	0.6
20c	M	0	1.2
21a	H	0.1	1
21b	H	0	0.6
21c	H	0	0.8
22a	H	0	0.9
22b	M	-0.1	1
22c	M	-0.1	0.7
23a	H	0	0.8
23b	H	0	0.6
23c	H	0	0.7

2 MBC-36 dataset

2.1 Structures

The coordinate files (*.xyz) of the MBC-36 complexes are available in the following Github repository: https://github.com/nlp-anh/CP-correction.git

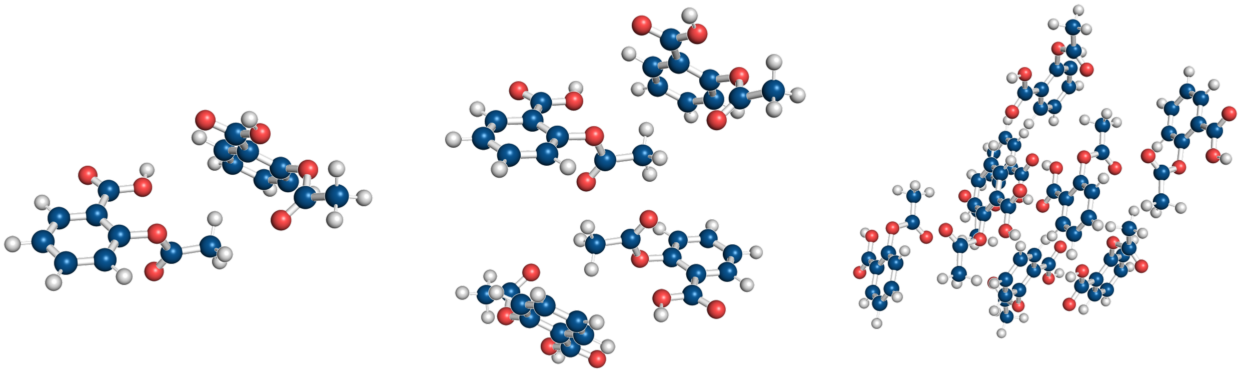

(a) Dimer **(b)** Tetramer **(c)** Octamer

Figure S1 Clusters of form I of Aspirin.

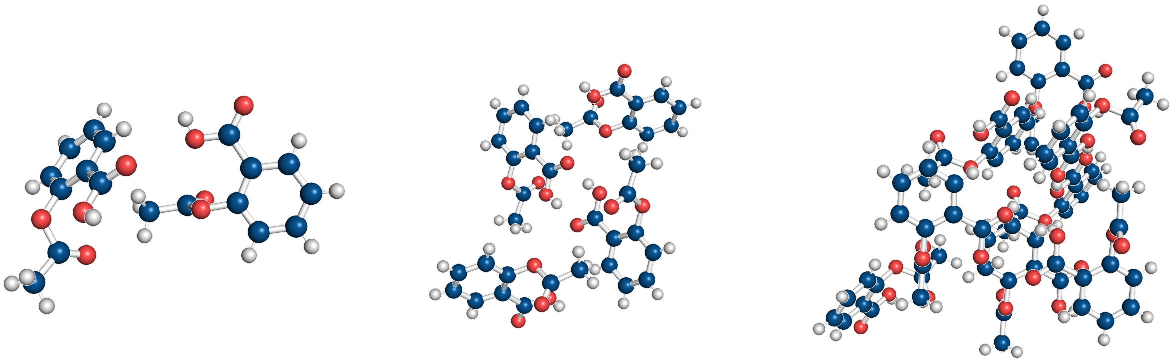

(a) Dimer **(b)** Tetramer **(c)** Octamer

Figure S2 Clusters of form II of Aspirin.

2.1.1 Aspirin

2.1.2 Benzene

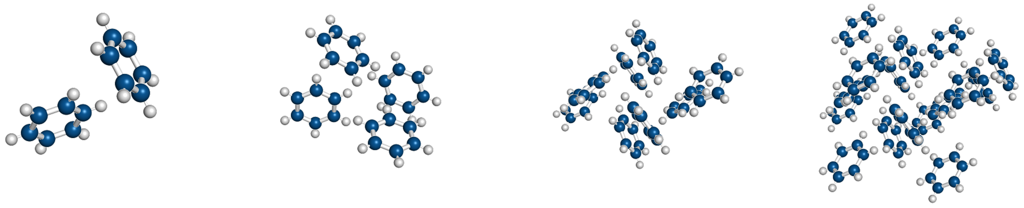

(a) Dimer **(b)** Tetramer **(c)** Octamer **(d)** Hexadecamer

Figure S3 Clusters of form I of Benzene.

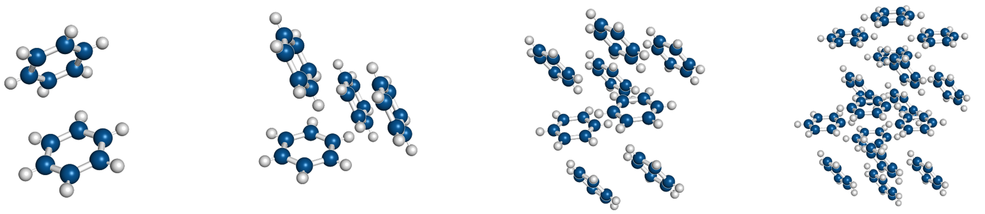

(a) Dimer **(b)** Tetramer **(c)** Octamer **(d)** Hexadecamer

Figure S4 Clusters of form II of Benzene.

2.1.3 Oxalyl dihydrazide

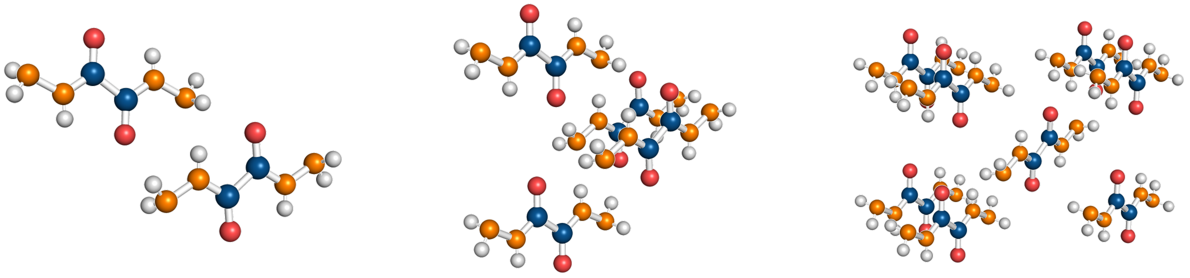

(a) Dimer **(b)** Tetramer **(c)** Octamer

Figure S5 Clusters of form α of Oxalyl Dihydrazide.

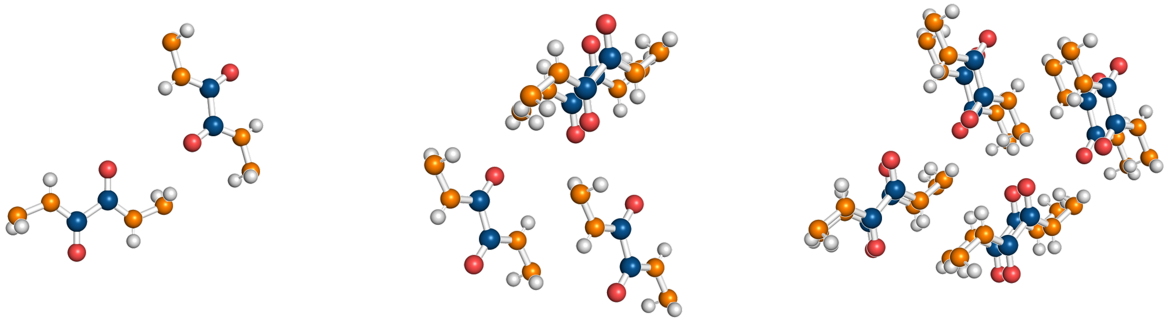

(a) Dimer **(b)** Tetramer **(c)** Octamer

Figure S6 Clusters of form β of Oxalyl Dihydrazide.

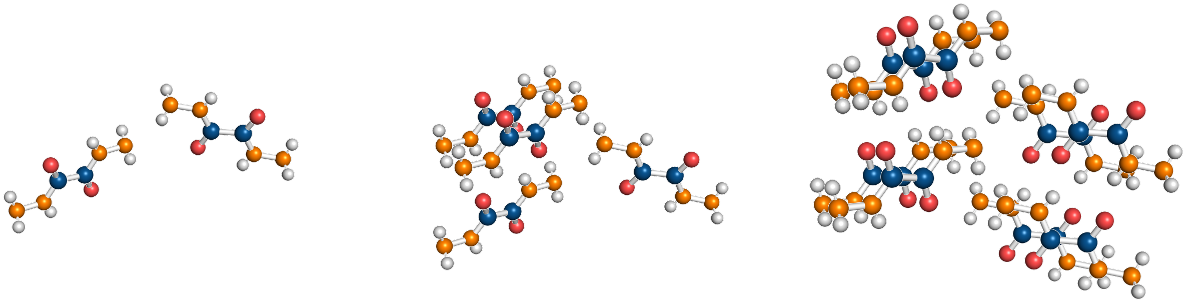

(a) Dimer **(b)** Tetramer **(c)** Octamer

Figure S7 Clusters of form δ of Oxalyl Dihydrazide.

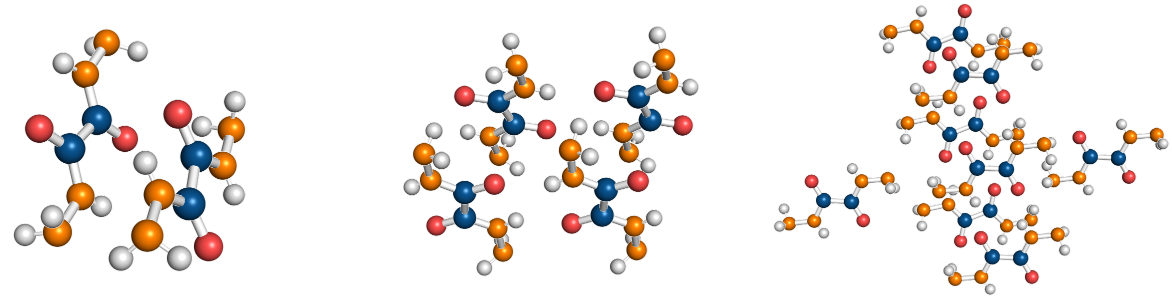

(a) Dimer. **(b)** Tetramer. **(c)** Octamer.

Figure S8 Clusters of form γ of Oxalyl Dihydrazide.

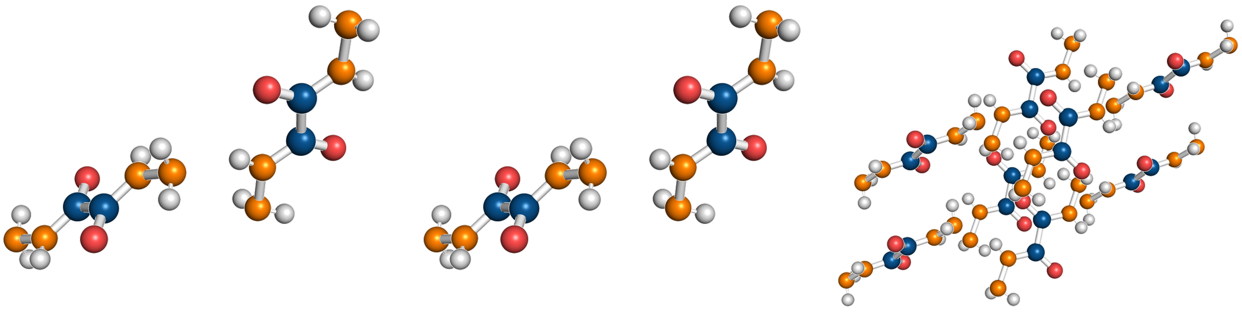

(a) Dimer **(b)** Tetramer **(c)** Octamer

Figure S9 Clusters of form ε of Oxalyl Dihydrazide.

2.2 Statistical errors

Table S6 Root mean square deviations (RMSD), mean unsigned errors (MUEs) and absolute maximum deviations (Max) (in kJ·mol^{-1}) for non-CP and CP-corrected HF/cc-pVDZ interaction energies of benzene clusters. All energies were calculated per molecule. CP-corrected HF/cc-pVQZ were taken as the benchmark.

ΔE^{INT}	Cluster size	RMSD	MUE	Max
CP	2	0.2	0.2	0.2
	4	0.1	0.1	0.1
	8	0.1	0.1	0.2
	16	0.2	0.1	0.2
non-CP	2	1.5	1.5	1.6
	4	3.1	3.1	3.3
	8	4.5	4.5	4.7
	16	5.8	5.8	6.5

Table S7 Root mean square deviations (RMSD), mean unsigned errors (MUEs) and absolute maximum deviations (Max) (in kJ·mol^{-1}) for non-CP and CP-corrected HF/cc-pVDZ interaction energies of aspirin clusters. All energies were calculated per molecule. CP-corrected HF/cc-pVQZ were taken as the benchmark.

ΔE^{INT}	Cluster size	RMSD	MUE	Max
CP	2	0.1	0.1	0.1
	4	0.3	0.2	0.4
	8	0.6	0.6	0.6
non-CP	2	3.4	3.4	3.6
	4	5.6	5.5	6.0
	8	12.3	12.3	13

Table S8 Root mean square deviations (RMSD), mean unsigned errors (MUEs) and absolute maximum deviations (Max) (in kJ·mol^{-1}) for non-CP and CP-corrected HF/cc-pVDZ interaction energies of oxalyl dihydrazide clusters. All energies were calculated per molecule. CP-corrected HF/cc-pVQZ were taken as the benchmark.

ΔE^{INT}	Cluster size	RMSD	MUE	Max
CP	2	0.1	0.1	0.2
	2	4.7	4.4	7.4
	4	0.1	0.1	0.2
non-CP	4	11.7	11.6	13.6
	8	0.4	0.3	0.6
	8	17.0	16.8	19.8

Table S9 Root mean square deviations (RMSD), mean unsigned errors (MUEs) and absolute maximum deviations (Max) (in kJ·mol^{-1}) for non-CP and CP-corrected HF/cc-pVTZ interaction energies of benzene clusters. All energies were calculated per molecule. CP-corrected HF/cc-pVQZ were taken as the benchmark.

ΔE^{INT}	Cluster size	RMSD	MUE	Max
CP	2	0.0	0.0	0.0
	4	0.0	0.0	0.0
	8	0.0	0.0	0.0
	16	0.0	0.0	0.1
non-CP	2	0.6	0.6	0.6
	4	1.2	1.2	1.3
	8	1.7	1.7	1.8
	16	2.2	2.2	2.4

Table S10 Root mean square deviations (RMSD), mean unsigned errors (MUEs) and absolute maximum deviations (Max) (in kJ·mol^{-1}) for non-CP and CP-corrected HF/cc-pVTZ interaction energies of aspirin clusters. All energies were calculated per molecule. CP-corrected HF/cc-pVQZ were taken as the benchmark.

ΔE^{INT}	Cluster size	RMSD	MUE	Max
CP	2	0.0	0.0	0.0
	4	0.1	0.1	0.1
	8	0.1	0.1	0.2
non-CP	2	1.3	1.3	1.4
	4	1.9	1.9	2.0
	8	4.3	4.3	4.5

Table S11 Root mean square deviations (RMSD), mean unsigned errors (MUEs) and absolute maximum deviations (Max) (in kJ·mol^{-1}) for non-CP and CP-corrected HF/cc-pVTZ interaction energies of oxalyl dihydrazide clusters. All energies were calculated per molecule. CP-corrected HF/cc-pVQZ were taken as the benchmark.

ΔE^{INT}	Cluster size	RMSD	MUE	Max
CP	2	0.1	0.1	0.1
	4	0.2	0.2	0.3
	8	0.2	0.2	0.3
non-CP	2	1.9	1.8	3.0
	4	4.4	4.4	4.9
	8	6.2	6.2	7.2

Table S12 Root mean square deviations (RMSD), mean unsigned errors (MUEs) and absolute maximum deviations (Max) (in kJ·mol^{-1}) for non-CP and CP-corrected HF/aug-cc-pVDZ interaction energies of benzene clusters. All energies were calculated per molecule. CP-corrected HF/cc-pVQZ were taken as the benchmark.

ΔE^{INT}	Cluster size	RMSD	MUE	Max
CP	2	0.0	0.0	0.0
	4	0.1	0.1	0.1
	8	0.1	0.1	0.1
	16	0.1	0.1	0.1
non-CP	2	2.8	2.8	2.9
	4	5.5	5.5	5.8
	8	8.7	8.7	8.9
	16	12.2	12.2	13.3

Table S13 Root mean square deviations (RMSD), mean unsigned errors (MUEs) and absolute maximum deviations (Max) (in kJ·mol^{-1}) for non-CP and CP-corrected HF/aug-cc-pVDZ interaction energies of aspirin clusters. All energies were calculated per molecule. CP-corrected HF/cc-pVQZ were taken as the benchmark.

ΔE^{INT}	Cluster size	RMSD	MUE	Max
CP	2	0.0	0.0	0.0
	4	0.1	0.1	0.2
	8	0.2	0.2	0.2
non-CP	2	3.3	3.2	3.4
	4	4.4	4.4	4.7
	8	11.2	11.1	12.6

Table S14 Root mean square deviations (RMSD), mean unsigned errors (MUEs) and absolute maximum deviations (Max) (in kJ·mol^{-1}) for non-CP and CP-corrected HF/aug-cc-pVDZ interaction energies of oxalyl dihydrazide clusters. All energies were calculated per molecule. CP-corrected HF/cc-pVQZ were taken as the benchmark.

ΔE^{INT}	Cluster size	RMSD	MUE	Max
CP	2	0.0	0.0	0.1
	4	0.1	0.1	0.2
	8	0.1	0.1	0.2
non-CP	2	2.0	1.8	3.2
	4	5.2	5.1	6.1
	8	8.1	8.0	9.0

Table S15 Root mean square deviations (RMSD), mean unsigned errors (MUEs) and absolute maximum deviations (Max) (in kJ·mol^{-1}) for non-CP and CP-corrected HF/aug-cc-pVTZ interaction energies of benzene clusters. All energies were calculated per molecule. CP-corrected HF/cc-pVQZ were taken as the benchmark.

ΔE^{INT}	Cluster size	RMSD	MUE	Max
CP	2	0.0	0.0	0.0
	4	0.0	0.0	0.0
	8	0.0	0.0	0.0
	16	0.0	0.0	0.0
non-CP	2	0.3	0.3	0.4
	4	0.6	0.6	0.6
	8	0.9	0.9	1.0
	16	1.2	1.2	1.4

Table S16 Root mean square deviations (RMSD), mean unsigned errors (MUEs) and absolute maximum deviations (Max) (in kJ·mol^{-1}) for non-CP and CP-corrected HF/aug-cc-pVTZ interaction energies of aspirin clusters. All energies were calculated per molecule. CP-corrected HF/cc-pVQZ were taken as the benchmark.

ΔE^{INT}	Cluster size	RMSD	MUE	Max
CP	2	0.0	0.0	0.0
	4	0.0	0.0	0.0
	8	0.0	0.0	0.0
non-CP	2	0.8	0.8	0.8
	4	1.1	1.1	1.2
	8	2.4	2.4	2.5

Table S17 Root mean square deviations (RMSD), mean unsigned errors (MUEs) and absolute maximum deviations (Max) (in kJ·mol^{-1}) for non-CP and CP-corrected HF/aug-cc-pVTZ interaction energies of oxalyl dihydrazide clusters. All energies were calculated per molecule. CP-corrected HF/cc-pVQZ were taken as the benchmark.

ΔE^{INT}	Cluster size	RMSD	MUE	Max
CP	2	0.0	0.0	0.0
	4	0.1	0.0	0.1
	8	0.1	0.0	0.1
non-CP	2	0.5	0.5	0.8
	4	1.5	1.4	1.9
	8	2.1	2.1	2.4

2.3 Raw deviations

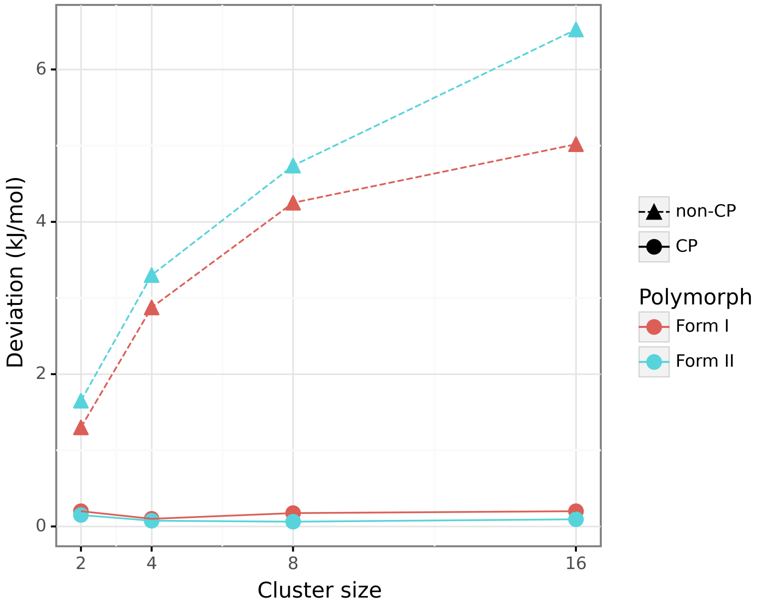

Figure S10 Behaviour of raw deviations (in kJ·mol^{-1}) of non-CP and CP-corrected HF/cc-pVDZ interaction energies with increasing cluster size for benzene clusters. CP-corrected HF/cc-pVQZ interaction energies were used as the benchmark.

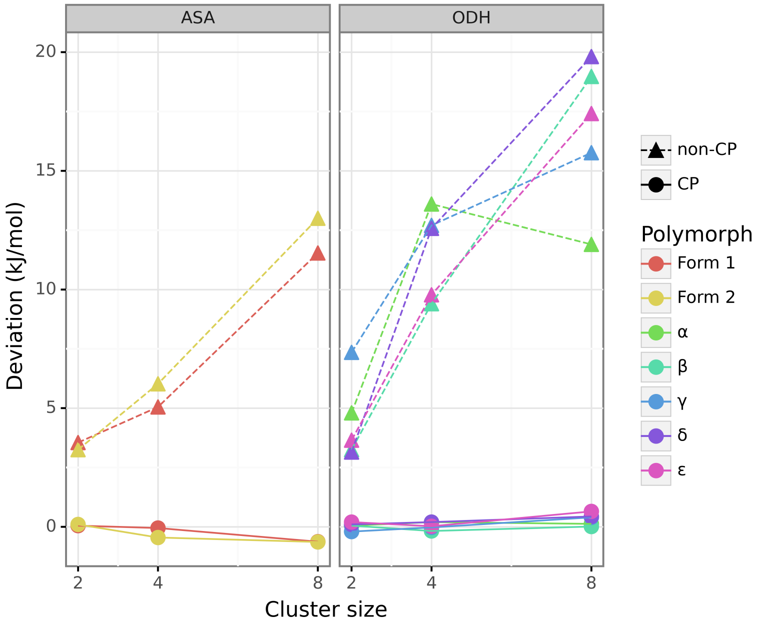

Figure S11 Behaviour of raw deviations (in kJ·mol^{-1}) of non-CP and CP-corrected HF/cc-pVDZ interaction energies with increasing cluster size for aspirin and oxalyl dihydrazide clusters. CP-corrected HF/cc-pVQZ interaction energies were used as the benchmark.

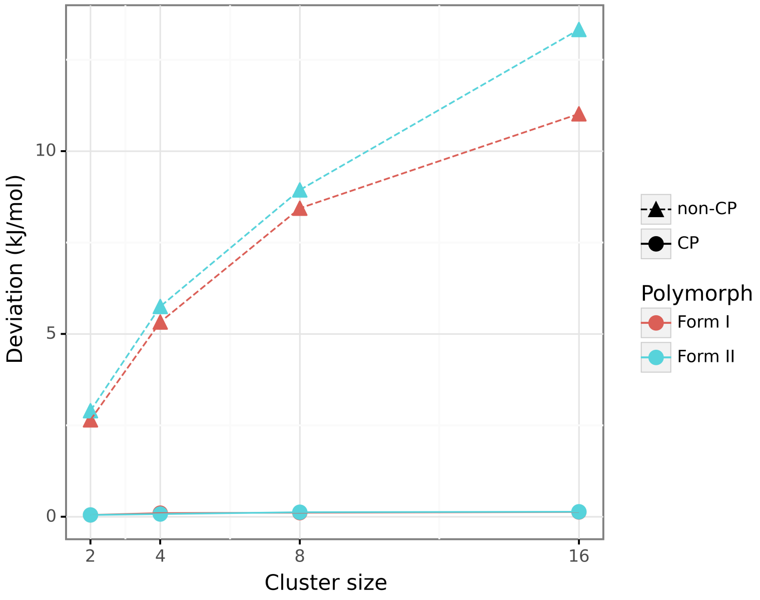

Figure S12 Behaviour of raw deviations (in kJ·mol^{-1}) of non-CP and CP-corrected HF/aug-cc-pVDZ interaction energies with increasing cluster size for benzene clusters. CP-corrected HF/cc-pVQZ interaction energies were used as the benchmark.

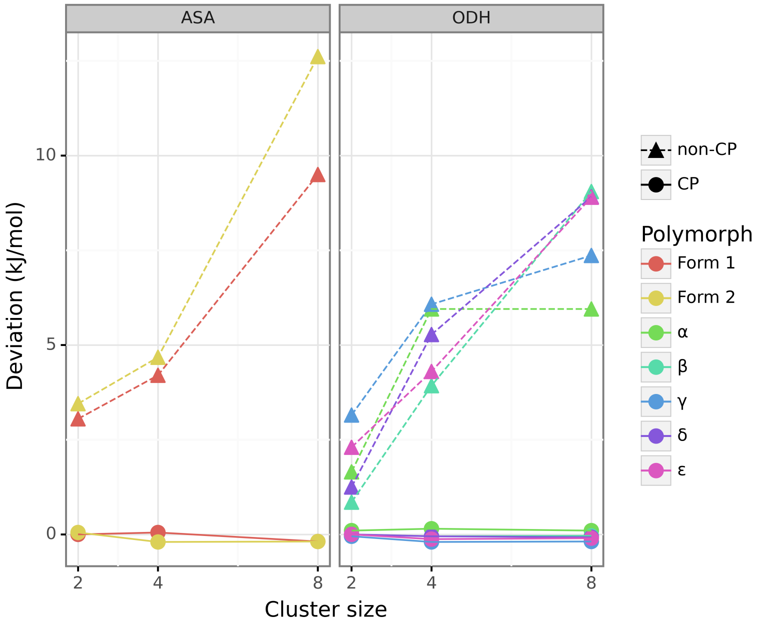

Figure S13 Behaviour of raw deviations (in kJ·mol^{-1}) of non-CP and CP-corrected HF/aug-cc-pVDZ interaction energies with increasing cluster size for aspirin and oxalyl dihydrazide clusters. CP-corrected HF/cc-pVQZ interaction energies were used as the benchmark.

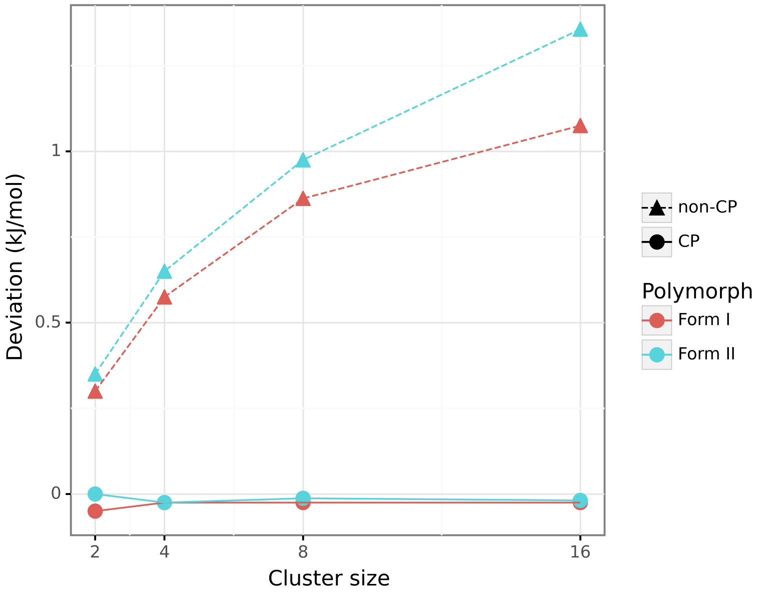

Figure S14 Behaviour of raw deviations (in kJ·mol^{-1}) of non-CP and CP-corrected HF/aug-cc-pVTZ interaction energies with increasing cluster size for benzene clusters. CP-corrected HF/cc-pVQZ interaction energies were used as the benchmark.

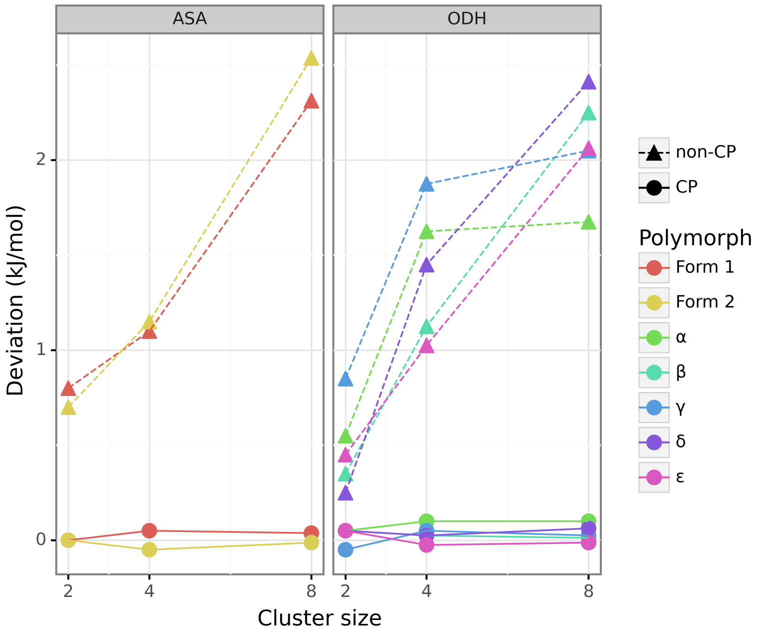

Figure S15 Behaviour of raw deviations (in kJ·mol^{-1}) of non-CP and CP-corrected HF/aug-cc-pVTZ interaction energies with increasing cluster size for aspirin and oxalyl dihydrazide clusters. CP-corrected HF/cc-pVQZ interaction energies were used as the benchmark.

3 CP correction with cut-off radius

Table S18 CP-corrected HF/cc-pVDZ energies of individual molecules in the 16-molecule benzene cluster (in Eh). The energies were calculated using the ghost molecules from the full system, from a sphere of 10 Å, 8 Å and 6 Å.

Molecule	Full	10 Å	8 Å	6 Å
1	−230.585749	−230.5857461	−230.5857426	−230.58537
2	−230.586327	−230.5863267	−230.586323	−230.58599
3	−230.585740	−230.5857374	−230.5857347	−230.58555
4	−230.585925	−230.5859242	−230.5859204	−230.58576
5	−230.585925	−230.5859245	−230.5859204	−230.58576
6	−230.586056	−230.5860529	−230.5860501	−230.58555
7	−230.586506	−230.5865051	−230.5865008	−230.58617
8	−230.586450	−230.586447	−230.5864435	−230.58595
9	−230.587603	−230.5876021	−230.5876007	−230.58723
10	−230.587610	−230.5876087	−230.5876073	−230.58723
11	−230.586358	−230.5863572	−230.5863547	−230.58603
12	−230.587571	−230.5875705	−230.5875664	−230.58719
13	−230.587894	−230.5878944	−230.5878924	−230.58737
14	−230.587346	−230.5873454	−230.5873382	−230.58641
15	−230.5873549	−230.5873549	−230.5873472	−230.58641
16	−230.586438	−230.5864374	−230.5864329	−230.58599

Table S19 CP-corrected HF/cc-pVTZ energies of individual molecules in the 16-molecule benzene cluster (in Eh). The energies were calculated using the ghost molecules from the full system, from a sphere of 10 Å, 8 Å and 6 Å.

Molecule	Full	10 Å	8 Å	6 Å
1	−230.663042	−230.6630396	−230.6630362	−230.66289
2	−230.663222	−230.6632212	−230.6632172	−230.66309
3	−230.663054	−230.6630517	−230.6630486	−230.66293
4	−230.663092	−230.6630897	−230.6630866	−230.66299
5	−230.663092	−230.6630901	−230.6630866	−230.66299
6	−230.663163	−230.6631611	−230.6631582	−230.66293
7	−230.663311	−230.6633096	−230.6633052	−230.66318
8	−230.663285	−230.6632835	−230.6632806	−230.66309
9	−230.663701	−230.6636999	−230.6636989	−230.6636
10	−230.663708	−230.6637075	−230.6637065	−230.6636
11	−230.663259	−230.6632584	−230.6632564	−230.66316
12	−230.663654	−230.6636541	−230.6636519	−230.66355
13	−230.663785	−230.6637854	−230.6637834	−230.66363
14	−230.663594	−230.6635938	−230.6635887	−230.66325
15	−230.6636045	−230.6636045	−230.6635992	−230.66325
16	−230.663263	−230.6632619	−230.6632559	−230.66309

Table S20 CP-corrected energies of individual molecules in the 2×2×2 supercells of form I of benzene calculated with cc-pVDZ and cc-pVTZ benzene cluster (in Eh). The energies were calculated using the ghost molecules from the sphere of 10 Åaround each corresponding molecule.

Molecule	cc-pVDZ	cc-pVTZ
1	−230.58575	−230.66304
2	−230.5868	−230.66344
3	−230.58633	−230.66322
4	−230.58638	−230.66327
5	−230.5876	−230.66368
6	−230.58669	−230.66335
7	−230.58574	−230.66305
8	−230.5866	−230.66336
9	−230.58592	−230.66309
10	−230.58646	−230.6633
11	−230.58766	−230.66375
12	−230.58667	−230.6634
13	−230.58737	−230.66361
14	−230.58869	−230.66402
15	−230.58737	−230.66361
16	−230.58667	−230.6634
17	−230.58766	−230.66375
18	−230.58646	−230.6633
19	−230.58592	−230.66309
20	−230.5866	−230.66336
21	−230.58574	−230.66305
22	−230.58669	−230.66335
23	−230.5876	−230.66368
24	−230.58638	−230.66327
25	−230.58633	−230.66322
26	−230.5868	−230.66344
27	−230.58575	−230.66304
28	−230.58763	−230.66372
29	−230.58763	−230.66372
30	−230.58866	−230.664
31	−230.58866	−230.664
32	−230.58764	−230.66373
33	−230.58763	−230.66373
34	−230.58763	−230.66373
35	−230.58764	−230.66373
36	−230.58866	−230.664
37	−230.58866	−230.664
38	−230.58763	−230.66372
39	−230.58763	−230.66372
40	−230.58758	−230.66367
41	−230.58759	−230.66367
42	−230.58758	−230.66367
43	−230.58759	−230.66367
44	−230.58867	−230.66401

Table S20 CP-corrected energies of individual molecules in the 2×2×2 supercells of form I of benzene calculated with cc-pVDZ and cc-pVTZ benzene cluster (in Eh). The energies were calculated using the ghost molecules from the sphere of 10 Åaround each corresponding molecule.

Molecule	cc-pVDZ	cc-pVTZ
45	−230.58867	−230.66401
46	−230.58867	−230.66401
47	−230.58867	−230.66401
48	−230.58759	−230.66367
49	−230.58758	−230.66367
50	−230.58759	−230.66367
51	−230.58758	−230.66367
52	−230.58735	−230.6636
53	−230.58868	−230.66401
54	−230.58736	−230.66361
55	−230.58735	−230.6636
56	−230.58868	−230.66401
57	−230.58736	−230.66361
58	−230.58736	−230.66361
59	−230.58868	−230.66401
60	−230.58735	−230.6636
61	−230.58736	−230.66361
62	−230.58868	−230.66401
63	−230.58735	−230.6636

Table S21 CP-corrected energies of individual molecules in the 2×2×2 supercells of form II of benzene calculated with cc-pVDZ and cc-pVTZ benzene cluster (in Eh). The energies were calculated using the ghost molecules from the sphere of 10 Åaround each corresponding molecule.

Molecule	cc-pVDZ	cc-pVTZ
1	−230.58959	−230.66554
2	−230.59019	−230.66576
3	−230.58962	−230.66553
4	−230.59093	−230.66596
5	−230.59181	−230.66626
6	−230.59049	−230.66584
7	−230.58994	−230.66565
8	−230.59025	−230.66577
9	−230.58943	−230.66548
10	−230.59014	−230.66573
11	−230.59092	−230.66598
12	−230.59041	−230.6658
13	−230.59139	−230.66609
14	−230.59263	−230.66643
15	−230.59139	−230.66609
16	−230.59041	−230.66581
17	−230.59092	−230.66598
18	−230.59014	−230.66573

Table S21 CP-corrected energies of individual molecules in the 2×2×2 supercells of form II of benzene calculated with cc-pVDZ and cc-pVTZ benzene cluster (in Eh). The energies were calculated using the ghost molecules from the sphere of 10 Åaround each corresponding molecule.

Molecule	cc-pVDZ	cc-pVTZ
19	−230.58943	−230.66548
20	−230.59025	−230.66577
21	−230.58994	−230.66565
22	−230.59049	−230.66584
23	−230.59181	−230.66626
24	−230.59093	−230.66596
25	−230.58962	−230.66553
26	−230.59019	−230.66576
27	−230.58959	−230.66554
28	−230.59144	−230.66616
29	−230.59144	−230.66616
30	−230.59142	−230.66616
31	−230.5914	−230.66615
32	−230.59227	−230.66636
33	−230.59228	−230.66636
34	−230.59228	−230.66636
35	−230.59227	−230.66636
36	−230.5914	−230.66615
37	−230.59141	−230.66616
38	−230.59144	−230.66616
39	−230.59144	−230.66616
